# The Importance of Spin State in Chiral Supramolecular Electronics

**DOI:** 10.3389/fchem.2021.722727

**Published:** 2021-08-04

**Authors:** Ana M. Garcia, Gabriel Martínez, Amparo Ruiz-Carretero

**Affiliations:** Institute Charles Sadron, University of Strasbourg, CNRS, Strasbourg, France

**Keywords:** supramolecular chirality, self-assembly, CISS effect, spin state, supramolecular electronics

## Abstract

The field of spintronics explores how magnetic fields can influence the properties of organic and inorganic materials by controlling their electron’s spins. In this sense, organic materials are very attractive since they have small spin-orbit coupling, allowing long-range spin-coherence over times and distances longer than in conventional metals or semiconductors. Usually, the small spin-orbit coupling means that organic materials cannot be used for spin injection, requiring ferromagnetic electrodes. However, chiral molecules have been demonstrated to behave as spin filters upon light illumination in the phenomenon described as chirality-induced spin selectivity (CISS) effect. This means that electrons of certain spin can go through chiral assemblies of molecules preferentially in one direction depending on their handedness. This is possible because the lack of inversion symmetry in chiral molecules couples with the electron’s spin and its linear momentum so the molecules transmit the one preferred spin. In this respect, chiral semiconductors have great potential in the field of organic electronics since when charge carriers are created, a preferred spin could be transmitted through a determined handedness structure. The exploration of the CISS effect in chiral supramolecular semiconductors could add greatly to the efforts made by the organic electronics community since charge recombination could be diminished and charge transport improved when the spins are preferentially guided in one specific direction. This review outlines the advances in supramolecular chiral semiconductors regarding their spin state and its influence on the final electronic properties.

## Introduction

**FIGURE 1:**
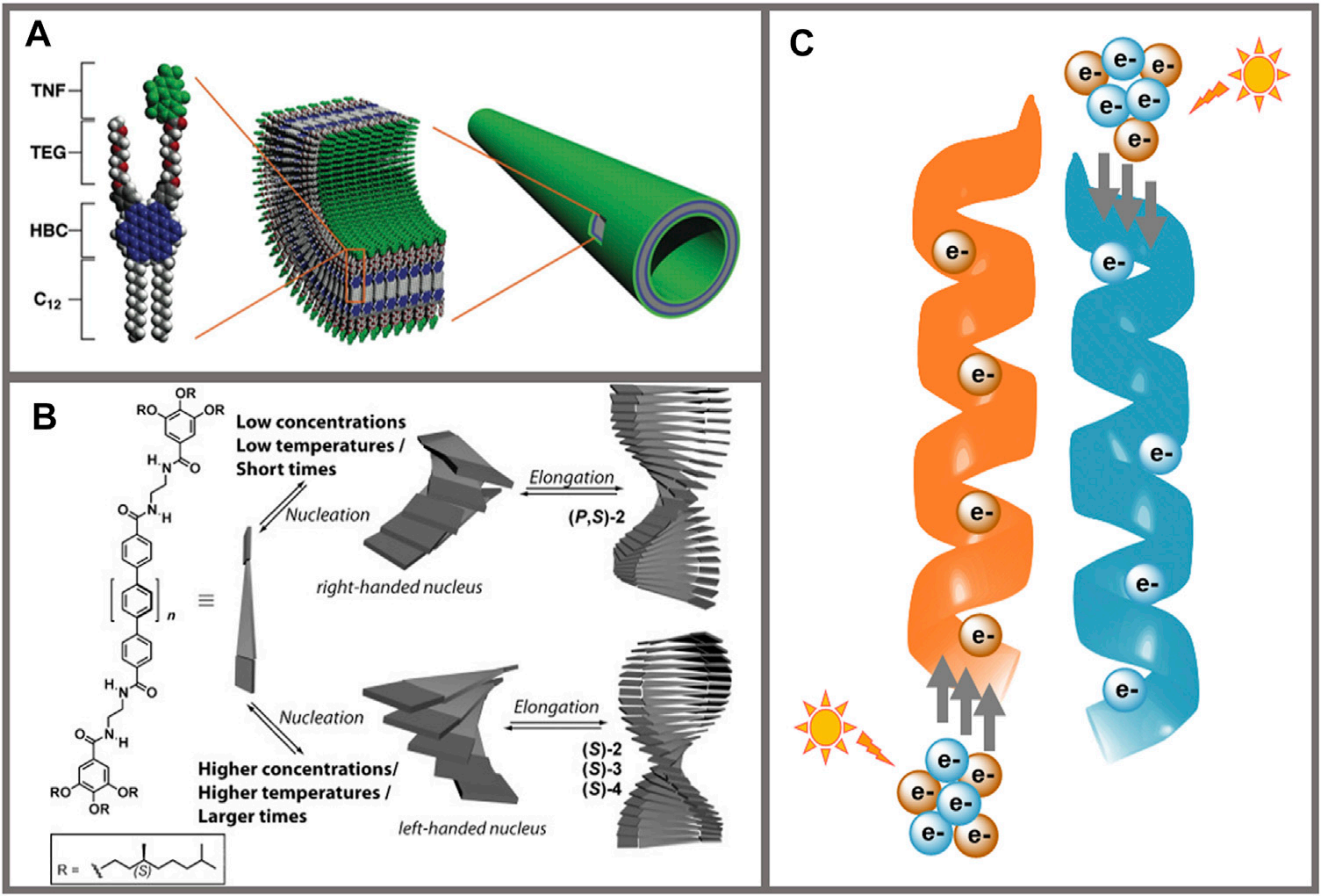
**(A)** Representation of the monomer (left) and the resulting supramolecular nanotube containing a coaxial p/n-heterojunction (right). The donor moiety in the monomer is HBC (blue), and the acceptor is trinitrofluorenone (green); TEG (red) and C12 chains (white) in the monomer provide solubility. From Yamamoto et al. 2006. Reprinted with permission from AAAS. **(B)** Schematic representation of the kinetically controlled modulation of the supramolecular helical organization of chiral oligo-p-phenylene-based organogelators. Structures of opposite handedness are obtained depending on the concentration, temperature, and times of formation. Reprinted with permission from Aparicio et al. (2014). Copyright 2014 Wiley-VCH. **(C)** Schematic representation of the CISS effect. The panel shows helical supramolecular structures where, after illumination with circular polarized light, electrons with opposite spins (orange and blue spheres) can be selectively transported through right-handed (orange) or left-handed (blue) helices, respectively.

The field of supramolecular electronics bridges the gap between molecular and plastic electronics (Meijer and Schenning, 2002; Schenning and Meijer, 2005; Moulin et al., 2013). In this sense, supramolecular chemistry represents the bridge, providing the tools to achieve highly organized structures with superior properties than those of the individual components. The presence of noncovalent interactions in organic semiconductors has been demonstrated to enhance the charge transport properties and device efficiency (Ghosh et al., 2017), finding exciting results in literature where π−π stacking interactions (Lee et al., 2011), hydrogen bonds (Huang et al., 2005; Ruiz-Carretero et al., 2013; Aytun et al., 2015; Carretero et al., 2020) metallophilic interactions (Che et al., 2011; Ruiz-Carretero et al., 2019) or a combination of several noncovalent interactions (Yamamoto et al., 2006; Schulze et al., 2014; Stupp and Palmer, 2014; Weldeab et al., 2018) were used to boost the properties of supramolecular electronic systems (Figure 1A). The dynamic nature of noncovalent interactions allows to tune the optoelectronic properties by controlling the self-assembly processes. In this regard, parameters such as temperature, concentration or solvent polarity can impact the self-assembly and hence, the properties (Aida et al., 2012). The incorporation of chiral centers into π-conjugated materials also affects the self-assembly properties. In this case, the chiral information of the monomer is transferred along the assembly yielding the final chiral configuration to the structure (Liu et al., 2015) (Figure 1B). Recently, chiral supramolecular structures have raised as very interesting systems in the field of spintronics (Wolf et al., 2001) since chiral and helical structures have been demonstrated to behave as spin filters upon light illumination in the phenomenon described as Chirality-Induced Spin Selectivity (CISS) effect (Naaman and Waldeck, 2012) (Figure 1C). Organic materials are very attractive for spintronic devices due to their small spin-orbit coupling (SOC), which increases the spin relaxation time as compared to inorganic materials usually containing heavy atoms, resulting in long-range spin transport in organic materials (Rocha et al., 2005). However, this affirmation should be taken carefully since the mobility of organic materials is rather inferior to those of inorganic materials, meaning that even if spin relaxation times are high, the spin polarized charges do not travel long distances (Szulczewski et al., 2009).

The small SOC implies that organic materials cannot be used for spin injection, requiring ferromagnetic electrodes (Awschalom and Flatté, 2007). However, the CISS effect indicates that organic molecules are not considered as passive elements but as spin filters because the lack of inversion symmetry in chiral molecules couples with the electron’s spin and its linear momentum, so the molecules transmit one preferred spin.

The scope of this review is to introduce the reader to chiral supramolecular electronic materials and the importance of the electron’s spin in the final properties of such materials. The CISS effect will be presented, as well as examples of supramolecular semiconductors where the roles of chirality and the spin have been highlighted but not related to the CISS effect. Finally, we focus on the latest insights into supramolecular systems based on chiral π-conjugated materials and the impact of controlling the spin state on the final electronic processes.

## The CISS Effect

### Discovery and Definition

The CISS effect was firstly identified by Naaman and coworkers in 1999, who determined the scattering asymmetry in electrons transmission in Langmuir-Blodgett films made of L- and D-stearoyl lysine (Ray et al., 1999). Their results showed that the quantum yield of photoelectrons depended both on the relative polarization of the light, and the chirality of the molecules. They started the study of the main factors that influence the CISS effect using self-assembled monolayers (SAMs) of double-stranded (ds) DNA and oligopeptides, demonstrating that spin selectivity is correlated to the supramolecular organization of single molecules, and its magnitude increases with the length of the DNA strands or peptide sequence, respectively (Carmeli et al., 2002; Ray et al., 2006; Göhler et al., 2011; Xie et al., 2011; Kettner et al., 2015; Aragonès et al., 2017; Kiran et al., 2017; Tassinari et al., 2018; Torres-Cavanillas et al., 2020). These studies pointed out the previously ignored role of the spin in electron-biomolecule interactions, as well as the potential of SAMs of chiral molecules that work as spin filters at room temperature (Michaeli et al., 2016; Naaman et al., 2019).

### Theoretical Models

Several theoretical models have been described to explain the spin-selective transport through chiral molecules, using helical-shaped molecules and dsDNA (Guo and Sun, 2012; Gutierrez et al., 2012; Medina et al., 2012). The first models found in literature share two important features: chirality is essential to reach spin polarization, and a helical potential based on the Rashba-like SOC term needs to be considered to calculate the SO interaction (Naaman and Waldeck, 2012). Later on, Dalum and Hedegard suggested a novel mechanism for CISS based on perturbative approach calculations, that need to be concretized to specific systems (Dalum and Hedegård, 2019). First-principle calculations were used by Gutierrez and coworkers to study the geometry-dependent spin polarization using an atomistic model of oligoglycine. The helical symmetry displayed a much higher spin polarization than the β-strand conformation, highlighting the role of helical geometry in the CISS effect (Maslyuk et al., 2018). In this sense, Herrmann et al. analyzed the crucial role of the imaginary terms in the Hamiltonian matrix for nonvanishing spin polarization in helical structures (Zöllner et al., 2020).

Furthermore, recent studies remark the important role of phonons and polarons to reach high spin polarization. Fransson showed the importance of cooperation of electron-phonon and spin-dependent couplings to get an exchange splitting between the spin channels that is reasonable for CISS (Fransson, 2020). In particular, he investigated systems of chiral molecules coupled to metals, where molecular vibrations (phonons) represent a mechanism able to break the spin symmetry of the molecule (Fransson, 2021). On their side, Zhang et al. assessed spin polaron transport in chiral molecules and, unlike previous theoretical explanations, their results showed that both type of polarons (spin-up and spin-down) can traverse the chiral molecule, although with different spin dynamics, i.e., the ones with antiparallel orientation experiment spin switching (Zhang et al., 2020).

Overall, there is not a general consensus as of now that theoretically rationalizes the astounding value experimentally observed for the CISS effect. Nevertheless, the investigations mentioned above suggest several reasons to explain this effect that range from the buildup of spin polarization at the interface to the electron-phonon interactions and polaron transport, and very recently, to the topological orbital texture combined with SOC in the substrate (Liu et al., 2021).Further theoretical investigations are currently ongoing that are expected to give more insights into these theoretical points.

### Experimental Measurements

There are multiple experimental methods to investigate the CISS effect (Naaman and Waldeck, 2015). Photoelectron spectroscopy has been used to characterize spin orientation through a SAM of chiral molecules adsorbed in a gold substrate when irradiating with circularly polarized light. The spin of the transmitted electrons is detected using a Mott polarimeter (Göhler et al., 2011). Conductive-probe atomic force microscopy (cp-AFM) is another technique that measures the spin-dependent conduction through single molecules (Xie et al., 2011; Kettner et al., 2015; Bullard et al., 2019). With this technique, the current-voltage (J-V) curves are registered on a SAM of chiral molecules adsorbed on a ferromagnetic substrate (normally nickel), while gold nanoparticles are attached to the tail of some of these molecules. It can be considered one of the best techniques to evaluate the real spin selectivity as it does not detect electrons from non-covered areas of the surface.

Spin polarization Hall devices measure Hall voltage and cyclic voltammetry response on chiral SAMs (Kumar et al., 2017; Bullard et al., 2019). The sign of the observed Hall voltage depends on the chirality of the molecule.

Recently, a technique that combines time-resolved microwave conductivity (TRMC), electron paramagnetic resonance (EPR) and optical spectroscopy has been used to study charge carrier mobility and spin state in p-type semiconductors (Tsutsui et al., 2018). Chemical doping using iodine vapors generates radicals that allow to determine the species with different spin state present in the sample.

## Chiral Supramolecular π-Conjugated Materials. Spin and Optoelectronic Properties

The importance of chirality and the spin state in organic electronics has been reported in many literature examples even if they were not connected to the CISS effect. Yet, the number of works linking conductivity to chirality is still scarce despite the emergent properties observed in organic semiconductors as a consequence of chirality (Yang et al., 2017). For instance, Zhu et al. reported optically active chiral electronic wires based on oligo-arylene-ethynylene and 1,1′-bi-2-naphthol (BINOL) (Zhu et al., 2006). The (R)- and (S)-derivatives were prepared and self-assembled onto gold surfaces. The electrical transport properties were studied measuring the J-V curves for the pure enantiomers and different enantiomeric mixtures, finding that the optically pure compounds exhibited greater conductivity than the mixtures. The authors hypothesized that the result could be due to very different packing structures between homochiral and heterochiral molecules. Later on, several works were reported on the influence of stereoisomerism on the crystallization, optoelectronic properties and device efficiency of π-conjugated materials functionalized with asymmetric branched alkyl chains. Liu et al. reported (Liu et al., 2013) the differences among the mesomer, the RR-isomer and the SS-isomer of diketopyrrolopyrrole (DPP) molecules functionalized with asymmetrical branched alkyl chains. The stereoisomers, isolated by a HPLC equipped with a chiral column, were also compared to the as-synthesized compound. The enantiomers showed very similar crystal structures, thin film morphology and field effect transistor (FET) properties, and they were the best structures to grow single crystals, while the mesomer had the greatest crystallization tendency in spin-cast films. The latter resulted in the highest charge carrier mobilities due to a coplanar conjugated backbone that favors intermolecular π−π stacking compared to the twisted backbone of the RR- and SS-isomers. Similarly, Zerdan et al. reported the influence of the solubilizing chain stereochemistry on photovoltaic devices made with small molecules and fullerene derivatives (Zerdan et al., 2014). In this case, the authors reported DPP derivatives with RR-, SS- and RS-ethylhexyl alkyl tails. Bulk heterojunction solar cells were fabricated with the pure isomers and compared to isomer mixtures from the purchased derivative. The authors found that when crystallization was induced by thermal annealing, important differences were found in the molecular packing between the different stereoisomers. Later on, Stolte et al. showed the impact of ethylhexyl stereoisomers on organic thin film transistors of π-conjugated materials (Stolte et al., 2016). In this case, the highest mobility is found for dyes bearing 2-ethylhexyl substituents that include a mixture of (R,R) (S,S) and (R,S) stereoisomers. The authors argue that this was possible due to the superior π−π contacts between DPP dyes. The result agreed with the previous studies pioneered by Liu and collaborators (Liu et al., 2013). The same group reported the impact of 2-ethylhexyl stereoisomers on single crystal field-effect transistors (FET) (He et al., 2018). In this case, the (R,S) mesomer was the most promising stereoisomer, being the mobility values superior to those of the pure enantiomers.

Other systems showing the influence of chirality in π-conjugated materials are optically active polymers (Grenier et al., 2007; Vanormelingen et al., 2008; Kane-Maguire and Wallace, 2010, 2010), thiophene-based block copolymers (Van den Bergh et al., 2010; Verswyvel et al., 2011), copolymers of chiral poly (ethylenedioxythiophene) (PEDOT) (Jeong and Akagi, 2011), supramolecular helical nanostructures (Hafner et al., 2018) and, tetrathiafulvalene systems (Pop et al., 2013, 2014).

Likewise, the role of the spin state was highlighted in other series of works. The spin state is a very important parameter in the kinetic control of recombination in organic photovoltaics (Rao et al., 2013) and in charge transfer (CT) states (Chang et al., 2015). While cascade structures allow the spatial separation of photogenerated electrons and holes in biological systems, the photogenerated excitons in organic photovoltaic devices are dissociated exclusively at the donor-acceptor heterojunction. However, the nanoscale morphology of photovoltaic devices promotes the encounters of charges and hence, recombination. Yet, there are examples of organic photovoltaic devices with quantum efficiency close to unity (Park et al., 2009), meaning that recombination can be avoided. Rao et al. (2013) demonstrated using time-resolved spectroscopy that the recombination of bound states is mediated not only by energetics, but also by the spin delocalization, allowing free carriers to be formed again and suppressing recombination. Along the same lines, Janssen et al. demonstrated that the spin-based particle reactions happening in polymer-fullerene blends can be tuned using magnetoresistance lineshapes and voltage dependencies (Janssen et al., 2013). The authors showed non-spin-polarized organic semiconductor devices, which in the absence of magnetic elements presented large room temperature magnetoresistance effect at small magnetic fields. This effect is known as organic magnetoresistance (OMAR) and it is very appealing because it can unravel unknown phenomena happening due to the intrinsically magnetic field-dependent charge transport properties of organic semiconductors. The authors explored the possible mechanisms to explain OMAR, categorized as reactions of polarons with the same charge into bipolarons, reactions of polarons with opposite charge into excitons, and reactions of triple excitons with polarons or with other triplet excitons. As a result of their study, the authors conclude that by choosing the right materials to alter the alignment of triplet excitons and CT states, important effects on the reaction pathways and the resulting OMAR can be achieved, influencing the device physics and efficiency.

## Main Supramolecular Structures Where CISS Effect has Been Studied

One of the main research areas of the CISS effect has been understanding its role in electron transfer in biology-related systems. It explains not only why it is so efficient in biological processes such as photosynthesis or respiration, but also the reasons for preferred enantioselective recognition in living organisms (Michaeli et al., 2016). In addition, other processes in which electrons are transferred in a way that only one spin state prevails are interesting for many applications in chemistry and electronics, since it enables the fabrication of electronic devices using chiral organic molecules instead of ferromagnets (Dor et al., 2013; Mathew et al., 2014; Koplovitz et al., 2017; Mtangi et al., 2017; Mondal et al., 2021).

In the next paragraphs we will describe the main supramolecular π-conjugated systems where the CISS effect has been studied.

### Peptides

**FIGURE 2:**
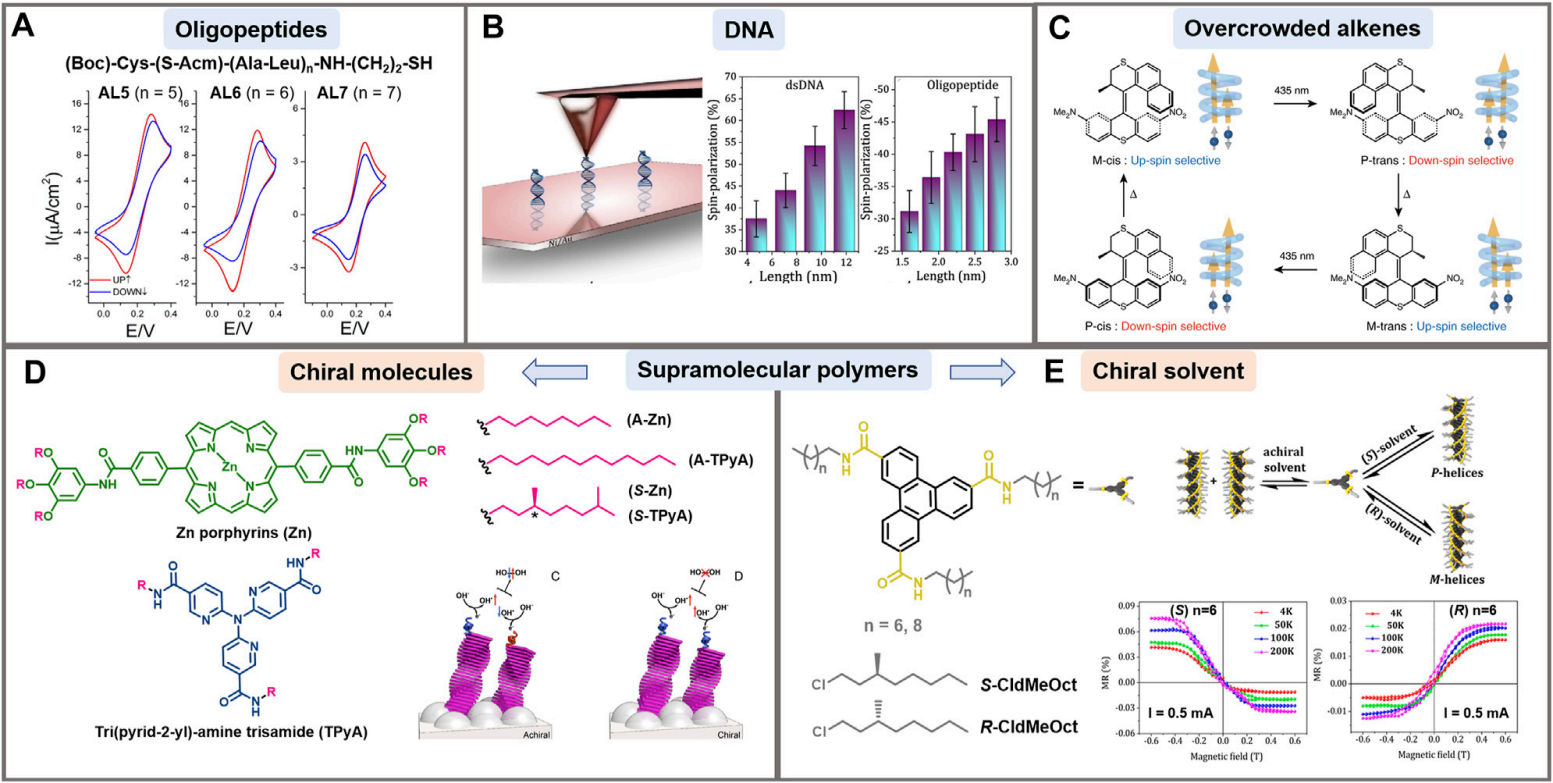
Examples of CISS effect in different supramolecular systems. **(A)** Cyclic voltammograms for the mM K_4_ [Fe(CN)_6_]/K_3_ [Fe(CN)_6_] redox couple in a 0.4 M KCl supporting electrolyte, aqueous solution. The working electrode is Ni covered with a self-assembled monolayer of oligopeptides AL5, AL6 and AL7, whose sequence is indicated. Red and blue curves indicate the two directions of magnetic field (conventionally up and down, respectively), which is normal to the surface of the working electrode. Reprinted with permission from Kettner et al. 2015. Copyright 2015 American Chemical Society. **(B)** Left: schematic representation of mc-AFM setup used to measure spin polarization in self-assembled monolayers of double stranded DNA. Right: Spin polarization results for various lengths of DNA and oligopeptides. Adapted with permission from Mishra et al. (2020a). Copyright 2020 American Chemical Society. **(C)** Schematic representation of the unidirectional rotation cycle of an overcrowded alkene driven by external stimuli. During the cycle, the chirality changes 4 times which results in a switch of the spin polarization direction of the electrons that are preferentially transported in the system. Reprinted with permission from Suda et al. 2019. Copyright 2019 Nature. **(D)** Supramolecular polymers where self-assembled helical structures (*M* or *P*) are formed by using chiral molecules in achiral solvents. The panel represents how coating the anode with the chiral molecules shown in the panel improves water splitting thanks to the CISS effect (vs. achiral ones), by avoiding the formation of hydrogen peroxide. Adapted with permission from Mtangi et al. 2017. Copyright 2017 American Chemical Society. **(E)** Supramolecular polymers where self-assembled helical structures (M or P) are formed from achiral molecules in the presence of chiral solvents. Adapted with permission from Mondal et al. 2021. Copyright 2021 American Chem Society.

Since the identification of the CISS effect, big efforts have been made to understand it in a wide variety of molecular systems, including biologically relevant molecules as DNA and peptides (Naaman and Waldeck, 2015). Only recently, several key parameters in spin polarization and its magnitude have been disclosed. The dependence of spin selectivity on the molecular length was demonstrated by varying the number of amino acid residues in oligopeptide sequences using cyclic voltammetry (Figure 2A) and cp-AFM (Kettner et al., 2015; Kiran et al., 2017), finding that spin selectivity decreases when increasing the tip-loading force. Following these studies, Aragonès and coworkers proved current asymmetry in chiral single molecular junctions by scanning tunneling microscopy break-junction (STM-BJ). They used 22-mer L- and D-oligopeptide systems, a magnetized nickel tip and a gold electrode (Aragonès et al., 2017). The spin selectivity in electron transfer was also noted when 12-mer oligopeptides were attached to ferrocene, where oxidation or reduction were favored depending on the *L*- or *D*-enantiomer and the direction of the magnetic field (Tassinari et al., 2018). Importantly, a polyproline chiral system (Pro_8_) conjugated to zinc porphyrins showed that the spin-polarized generated currents were further transmitted over distances surpassing the length of chiral molecules (Bullard et al., 2019). Very recently, ds peptide nucleic acids (PNAs) have been proposed to study the CISS effect. Their spin-filtering capabilities were directly correlated to the molecular helicity, highlighting the worth of the dsPNAs for systematic studies of the CISS effect (Möllers et al., 2021).

### DNA

The helical structure of dsDNA is very attractive in spintronics because it has played a critical role in charge transport processes through long molecular distances (Kiran et al., 2017). In 2011, Göhler and collaborators presented the first example of spin filters based on DNA. They obtained spin polarization exceeding 60% at room temperature measured by Mott polarimetry with dsDNA monolayers adsorbed on gold (Göhler et al., 2011). Densely packed single stranded (ss) and dsDNA films with a redox-active probe adsorbed on a gold-capped nickel surface were analyzed by cyclic voltammetry. Only the dsDNA films displayed variations of up to 16% in the electrochemical reduction depending on the orientation of the magnetic field. This states that the chiral supramolecular organization prevails over the chirality of the individual components (Zwang et al., 2016). The linear dependence of the spin polarization on the length of dsDNA oligonucleotides has also been demonstrated by cp-AFM using lengths of 20 up to 50 base pairs (Mishra et al., 2020a) (Figure 2B). Later, Banerjee-Ghosh et al. proved experimentally that there is an enantiospecific interaction between chiral molecules and perpendicularly polarized substrates. They followed the kinetics of the enantioselective adsorption of dsDNA on a magnetized Ni/Au surface, finding that the rate of absorption was considerably different for up and down magnetization of the substrate (Banerjee-Ghosh et al., 2018). Additionally, researchers have investigated the effect of oxidative damage on the spin transport through monolayers of dsDNA using a Hall device (Bullard et al., 2019). Unexpectedly, dsDNA having one and two oxidative damages in the base pairs had higher spin polarization than undamaged dsDNA films. It seems that due to the damage of the bases most of the conduction goes through the backbone of the DNA structure, which is chiral and hence, more spin selective.

### Helicenes and Overcrowded Alkenes

Helicenes are fully conjugated molecules without stereogenic carbons. The repulsion between the termini of these molecules makes the helicenes adopt permanent helical conformations with *M* (left-handed enantiomer) and *P* (right-handed enantiomer) configurations (OuYang and Crassous, 2018). Kiran et al. have shown that cationic [4] helicenes behaved as spin filters when they were uniformly absorbed and oriented on a pyrolytic graphite surface (Kiran et al., 2016). Spin polarizations of more than 40% were obtained with preferred opposite spin orientation for *P* and *M* configurations. In another report, monolayers made of enantiopure [7] helicenes were deposited on Cu (332), Ag (110), and Au (111) surfaces, which have a wide range of SOC values (Kettner et al., 2018). Very similar results of spin selectivity were obtained, proving the dominant role of chirality in the spin filtering ability of helicenes over the SOC of the surfaces. Interestingly, some authors have pointed out improved charge transport properties on racemic mixtures of helicenes compared to enantiopure composition in organic electronic devices (Yang et al., 2017). Important morphological differences between the racemic and enantiopure systems were found, as well as an 80-fold increase in hole mobility in FETs. On the other hand, Josse et al. compared device efficiency fabricated with enantiopure and racemic naphthalimide end-capped [6] helicenes as electron acceptors (Josse et al., 2017), observing a two-fold increase in electron mobility, and a five-fold increase of the power conversion efficiency in devices fabricated with the enantiopure material compared to the racemic. These contradictory results emphasize the need to further investigate the impact of solid-state organization in chiral supramolecular systems in organic electronic devices. In 2019, it was presented for the first time the change of spin selectivity by modifying the handedness of chiral molecules by external stimuli (Suda et al., 2019) (Figure 2C). An artificial molecular motor based on an overcrowded alkene was synthesized. It was able to switch its chirality generating a unidirectional rotation cycle driven by temperature or light, with spin selectivity values of up to 44%.

### Conjugated Polymers/π-Conjugated Molecules Incorporating Amino Acids (Chirality)

Chirality has been demonstrated crucial for spin filtering also in different polymers and π-conjugated molecules, as for example in organic light emitting diodes (OLEDs). Thanks to the CISS effect, chiral polymers represent a great alternative for spin polarization and injection with high spin selectivity. For instance, thin films of thiophene-based polymers incorporating cysteine exhibited high spin filter ability at room temperature, as shown using a solid-state device to determine magnetoresistance and electrochemical measurements (Mondal et al., 2015). Another intriguing example illustrating the importance of selective spin transport in supramolecular structures is the improvement in water splitting by avoiding the formation of hydrogen peroxide (Figure 2D). In this case, the anode was coated with a helix-forming chiral organic semiconductor that enhanced the desired process thanks to the CISS effect (Mtangi et al., 2017). Later on, the importance of supramolecular chirality rather than the number of chiral centers present in the molecule was demonstrated using coronene bisimide and porphyrin-like polymers with chiral (or achiral) alcoxyphenyl chains (Kulkarni et al., 2020). In principle, supramolecular helicity is expected to be inverted depending on the stereoconfiguration of the chiral centers in the π-conjugated molecule or polymer. However, it was shown that both, *M* and *P* chiral helicity can also emerge from a monomer with the same chirality (e.g., *L*-derivative). In this sense, the secondary arrangement can be inverted by changing the temperature (+20°C or −10°C) (Kulkarni et al., 2020), or by using a different solvent (Mishra et al., 2020b). More recently, spin polarization was identified in achiral polymers with a preferred helical arrangement induced by the use of chiral solvents. The authors used triphenylene-2,4,10-tricarboxamide derivatives, whose supramolecular chirality is biased to get either *P*- or *M*-helices when using chiral solvents (Mondal et al., 2021) (Figure 2E). The inversion of supramolecular chirality by means of temperature and solvent when using the same enantiomer affects spin selectivity, and confirms the importance of supramolecular orientation in selective spin transport. In fact, very recently, Meijer and coworkers claimed the pivotal role of chiral supramolecular order rather than the number of chiral centers in discrete molecules in the CISS effect using squarine dyes (Rösch et al., 2021).

### Inorganic and Hybrid Inorganic-Organic Materials

Inorganic and hybrid inorganic-organic materials have shown as well properties as spin filters. Hybrid materials of perovskites frameworks integrating a chiral organic sublattice have presented spin selectivity much larger than previously reported in SAM systems (Waldeck et al., 2021). Recently, Lu et al. achieved spin polarizations of up to 86% in oriented R- and S-chiral 2D-layered Pb-iodide hybrid organic-inorganic perovskite (HOIP) films. Weak thickness dependence was displayed in films from 20–100 nm (Lu et al., 2019) In another report, Huang et al. demonstrated that chiral-HOIPs are capable of changing the magnetization of an adjacent NiFe ferromagnetic substrate. The sign of the magnetization studied by Magneto-optic Kerr rotation effect depended on the chirality of the HOIP (Huang et al., 2020). Recently, a spin-polarized LED at room temperature without magnetic or ferromagnetic contacts, which are normally required has been reported (Kim et al., 2021). Furthermore, bioinspired chiral metal-organic Cu(II) phenylalanine (D- or L-) crystals have shown to present CISS electron conduction over long ranges (300 nm) at room temperature measured by magnetic cp-AFM. Interestingly, the authors also reported a thermally activated ferromagnetic behavior, which had only been identified in inorganic materials(Huang et al., 2020).

In 2019 Ghosh et al. prepared copper oxide films capable of spin polarize photoelectrons and act as electrocatalyst for the conversion of water to oxygen. The spin filtering ability of chiral CuO avoids the generation of side products such as H_2_O_2_ (Ghosh et al., 2019). In another example by the same group, chiral cobalt oxide films used as electrocatalysts in the oxygen evolution reaction achieved a 1.4-fold increase in the production of oxygen(Ghosh et al., 2020).

## Future Directions in the Field

The CISS effect has been identified and studied in many different systems, especially over the last 20 years. Although it is still at its infancy, experimental studies of this phenomenon and the attempts to give an accurate theoretical explanation have paved the way for a better understanding of the effect itself. Over the next years, its application in the fabrication and the development of novel devices is expected, where miniaturization and reduction of energy consumption can be envisaged, as the use of ferromagnets, and more complicated interfaces can be avoided. The goal is to achieve the proper supramolecular organization to ensure spin polarization and filtering, either using pure chiral entities or in combination with achiral molecules where supramolecular chirality can be achieved as described by the “sergeants-and-soldiers” effect. Overall, the study and application of the CISS effect can revolutionize spin-based devices in the organic electronics field.
